# Left Atrial Volumes and Strains in Healthy Mid-Term Pregnancy—A Detailed Investigation from a Three-Dimensional Speckle-Tracking Echocardiographic MAGYAR-Preg Study

**DOI:** 10.3390/biomedicines14061225

**Published:** 2026-05-29

**Authors:** Attila Nemes, Renáta Halcsik, Árpád Kormányos, Nándor Gyenes, Kitti Rajcsány, Barbara Bordács, Nóra Ambrus, Mohammad Nasiri, Csaba Lengyel, Tibor Novák

**Affiliations:** 1 Department of Medicine, Albert Szent-Györgyi Medical School, University of Szeged, 6725 Szeged, Hungary; halcsik.renata@med.u-szeged.hu (R.H.); kormanyos.arpad@med.u-szeged.hu (Á.K.); gyenes.nandor@med.u-szeged.hu (N.G.); rajcsany.kitti@med.u-szeged.hu (K.R.); bordacs.barbara.aniko@med.u-szeged.hu (B.B.); ambrusnora@gmail.com (N.A.); lengyel.csaba@med.u-szeged.hu (C.L.); 2 Department of Obstetrics and Gynaecology, Albert Szent-Györgyi Medical School, University of Szeged, 6720 Szeged, Hungary; nasiri.mohammad@med.u-szeged.hu (M.N.); novak.tibor@med.u-szeged.hu (T.N.)

**Keywords:** healthy, pregnancy, left atrial, strain, three-dimensional, echocardiography

## Abstract

**Introduction**: Gestational physiology is characterized by an expansion of plasma volume and an elevation in cardiac output. Given the scarcity of existing data on pregnancy-related left atrial (LA) volumetric and functional features, this study aims to define LA volumes, volume-based functional properties and strains in healthy subjects during mid-term pregnancy. **Methods**: The present study comprised 19 healthy women in mid-term pregnancy (mean age: 30.5 ± 2.7 years, weight: 81.7 ± 14.0 kg, height: 166.9 ± 5.7 cm) without any symptoms, known diseases or other conditions, which could affect the results. Their results were compared to those of 43 healthy non-pregnant women (mean age: 28.6 ± 4.9 years, weight: 59.9 ± 8.5 kg, height: 167.8 ± 7.6 cm). All participants underwent comprehensive two-dimensional Doppler echocardiography with three-dimensional speckle-tracking echocardiography (3DSTE). **Results**: Thicker interventricular septum, increased left ventricular ejection fraction and impaired early and late transmitral flow velocities could be detected in healthy pregnant subjects as compared to those of non-pregnant individuals. End-systolic maximum LA volume was increased with elevated stroke volume and emptying fraction. While early diastolic LA volume was preserved with elevated stroke volume and emptying fraction, late diastolic LA volume, stroke volume and emptying fraction remained unchanged. However, indexed LA volumes did not differ between the groups. Among end-systolic peak global LA strains, only LA longitudinal strain (LS) was increased, while all others remained unchanged. Among regional strains, basal, midatrial and superior LA circumferential strain (CS) and LA-LS were increased except for basal LA-CS, which was impaired. Among late diastolic LA strains at atrial contraction, none of them showed any significant changes in healthy pregnant subjects compared with those of non-pregnant women. **Conclusions**: With a detailed 3DSTE study, elevated end-systolic LA volume and preserved diastolic LA volumes, together with enhanced end-systolic LA reservoir and early diastolic LA conduit functional properties, could be detected with features of preserved late diastolic booster pump function in healthy women during mid-term pregnancy (second trimester). When comparing indexed LA volumes, no significant difference could be confirmed between the pregnant and non-pregnant groups. This suggests that the increased end-systolic LA volume may be an adaptation to increased body weight.

## 1. Introduction

Gestational physiology is characterized by an expansion of plasma volume and an elevation in cardiac output, necessitating substantial structural and functional remodeling of the cardiac chambers [1]. The left atrium (LA) is a critical component of the central circulation, exhibiting dynamic phasic activity throughout the cardiac cycle with corresponding changes in volume and myocardial contractility [2,3,4]. Given the extensive physiological adaptations associated with pregnancy, it is essential to characterize the longitudinal changes in LA morphology and function in healthy maternal and fetal conditions. Modern imaging techniques appear suitable for the non-invasive volumetric and functional analysis of the LA. Among these methods, three-dimensional (3D) speckle-tracking echocardiography (3DSTE) stands out as a user-friendly and easily learnable tool, validated for the simultaneous evaluation of both volumetric and functional LA changes. By generating a virtual 3D model from a single dataset, this technique measures specific parameters throughout the cardiac cycle to assess atrial health [5,6,7,8]. Given the scarcity of existing data on the pregnancy-related LA volumetric and functional features, this study aims to define LA volumes, volume-based functional properties and strains in healthy subjects during mid-term pregnancy and to compare their findings to those of adult non-pregnant females.

## 2. Materials and Methods

### 2.1. Study Population

The present study comprised 19 healthy women in mid-term pregnancy (mean age: 30.5 ± 2.7 years, weight: 81.7 ± 14.0 kg, height: 166.9 ± 5.7 cm, the mean gestational age was 22.0 ± 5.6 weeks) without any symptoms, known diseases or other conditions that could affect the results. Their findings were compared to those of age-matched 43 healthy non-pregnant women (mean age: 28.6 ± 4.9 years, weight: 59.9 ± 8.5 kg, height: 167.8 ± 7.6 cm). The Mosteller formula is used to estimate body surface area (BSA); it is defined as the square root of the product of weight (kg) and height (cm) divided by 3600 [9]. None of the women received any medication at the time of the examination or were smokers or athletes. All participants underwent comprehensive two-dimensional (2D) Doppler echocardiography and 3DSTE. This retrospective cohort study is part of the **MAGYAR-Preg Study** (**M**otion **A**nalysis of the heart and **G**reat vessels b**Y** three-dimension**A**l speckle-t**R**acking echocardiography in **Preg**nancy). This ongoing research initiative, conducted at the University of Szeged, aims to characterize pregnancy-related myocardial and valvular abnormalities using 3DSTE. This study’s name incorporates the word “Magyar”, which translates to “Hungarian” in the Hungarian language. The research protocol adheres to the 2013 revision of the Declaration of Helsinki and has been formally approved by the Institutional and Regional Human Biomedical Research Committee of the University of Szeged under reference numbers 71/2011 (with latest approval on 17 March 2025) and 145/2021 (with latest approval on 17 June 2024). All participants provided written informed consent prior to inclusion, and this study was conducted in accordance with the stated approvals and consent procedures.

### 2.2. Two-Dimensional Doppler Echocardiography

2D Doppler echo was performed in all subjects by experienced operators using a Toshiba Artida™ echocardiography system (Toshiba Medical Systems, Tokyo, Japan) equipped with a PST-30BT (1–5 MHz) phased-array transducer. Chamber quantifications were conducted in accordance with current professional guidelines and routine clinical practice. Doppler echocardiography was utilized to detect valvular regurgitation and stenosis, as well as to determine early (E) and late (A) diastolic mitral inflow velocities [9].

### 2.3. Three-Dimensional Speckle-Tracking Echocardiography

3DSTE data acquisition was performed using the Toshiba Artida™ system (Toshiba Medical Systems, Tokyo, Japan) equipped with a PST-25SX matrix array transducer [5,6,7,8]. Volumetric 3D datasets were digitally acquired from the apical window during a single breath-hold. In all individuals in sinus rhythm, six wedge-shaped subvolumes were obtained over six consecutive cardiac cycles to automatically generate a full-volume pyramidal dataset. Offline analysis was conducted using the 3D Wall Motion Tracking software (version 2.7, Toshiba Medical Systems, Tokyo, Japan). The 3D datasets were displayed in apical four-chamber and two-chamber long-axis views, as well as in three short-axis views (basal, midatrial, and superior LA regions). Following the selection of several reference points, the endocardial surface was traced starting from the mitral annular plane and proceeding around the LA, ensuring the exclusion of the LA appendage and pulmonary veins. The software then performed automatic 3D wall motion tracking from the end-diastolic reference frame, with manual adjustments permitted throughout the analysis.

### 2.4. 3DSTE-Derived LA Quantifications

**Figure 1:**
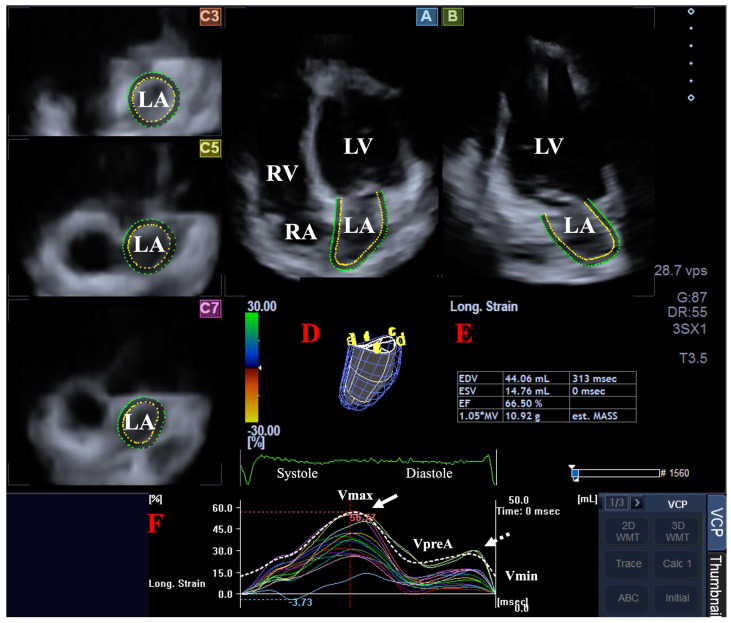
Three-dimensional (3D) speckle-tracking echocardiographic (3DSTE) assessment of the left atrium (LA) in a healthy subject using a full-volume dataset is demonstrated. The analysis incorporates apical four-chamber (**A**) and two-chamber (**B**) views, as well as short-axis views at the basal (**C3**), midatrial (**C5**), and superior (**C7**) levels to create a comprehensive 3D LA cast (**D**). LA volumetric data (**E**) are presented alongside with time-dependent curves for global LA volume change (represented by a dashed line) and segmental LA longitudinal strains (represented by colored lines) (**F**). In the visualization, the white arrow indicates peak LA strains, while the white dotted arrow marks LA strains specifically at atrial contraction. **Abbreviations.** LA, left atrium; LV, left ventricle; RA, right atrium; RV, right ventricle; EDV, end-diastolic volume; ESV, end-systolic volume; EF, ejection fraction; Vmax, maximum LA volume; VpreA, pre-atrial contraction LA volume; Vmin, minimum volume.

LA volumes were determined at specific phases of the cardiac cycle using the aforementioned 3D LA model (Figure 1). These included the end-systolic maximum LA volume (Vmax, measured at mitral valve opening), the LA volume at the onset of atrial contraction (VpreA, corresponding to the P-wave on ECG), and the end-diastolic minimum LA volume (Vmin, measured at mitral valve closure). Based on these volumetric data, the following LA functional parameters were calculated [5,6,7,8]:

- Systolic reservoir function: represented by the LA total stroke volume (TASV = Vmax − Vmin) and the LA total emptying fraction (TAEF = TASV/Vmax).
- Early diastolic conduit function: characterized by the LA passive stroke volume (PASV = Vmax − VpreA) and the LA passive emptying fraction (PAEF = PASV/Vmax).
- Late diastolic booster pump function: defined by the LA active stroke volume (AASV = VpreA − Vmin) and the LA active emptying fraction (AAEF = AASV/VpreA).

All measurements were averaged over three consecutive cardiac cycles. Using the same 3D virtual model, several strain parameters were automatically calculated: unidirectional radial (RS), longitudinal (LS), and circumferential (CS) strains, as well as multidimensional complex area (AS) and 3D (3DS) strains. RS reflects the thinning/thickening, LS reflects the lengthening/shortening, and CS reflects the widening/narrowing of the LA segments. AS represents the combination of LS and CS, while 3DS incorporates RS, LS, and CS. The 3DSTE-derived LA strain curves display two peaks: the first corresponds to end-systolic reservoir function, while the second reflects end-diastolic atrial contraction (LA systole, booster pump function). Global and segmental strains were automatically generated by the software. Regional strains were calculated by averaging the segmental data within each respective region, while mean segmental strains were derived from all segments.

### 2.5. Statistical Analysis

Continuous variables are expressed as the mean ± standard deviation (SD), while categorical data are presented as frequencies and percentages, as appropriate. A *p*-value < 0.05 was defined as the threshold for statistical significance. Data distribution was assessed for normality using the Shapiro–Wilk test, and homogeneity of variances was evaluated via Levene’s test. For normally distributed data, Student’s *t*-test was employed, whereas the Mann–Whitney U test was used for non-parametrically distributed datasets. Categorical variables were compared using Fisher’s exact test. To evaluate intra- and interobserver variability for LA parameters, intraclass correlation coefficients (ICCs) were calculated in 25 randomly selected healthy individuals. Based on the final sample size, a 0.05 significance level, and the observed effect size, a retrospective power analysis was performed using G*Power 3.1. All statistical analyses were conducted using SPSS software (version 22.0; IBM SPSS Inc., Chicago, IL, USA).

## 3. Results

### 3.1. Two-Dimensional Doppler Echocardiography

Thicker interventricular septum, increased LV ejection fraction and impaired early and late transmitral flow velocities could be detected in healthy pregnant subjects as compared to those of non-pregnant individuals (Table 1). None of the subjects showed ≥grade 1 valvular regurgitation or significant valvular stenosis.

### 3.2. 3DSTE-Derived LA Volumes

The frame rate proved to be 30 ± 3 vps. End-systolic maximum LA volume (Vmax) was increased with elevated stroke volume (TASV) and emptying fraction (TAEF). While early diastolic LA volume (VpreA) proved to be preserved with elevated stroke volume (PASV) and emptying fraction (PAEF), late diastolic LA volume (Vmin), stroke volume (AASV) and emptying fraction (AAEF) remained unchanged. When index LA volumes were compared, no differences between groups could be detected (Table 2).

### 3.3. 3DSTE-Derived LA Strains

From end-systolic peak global LA strains, only LA-LS (and consequential LA-AS) proved to be increased; all others remained unchanged. From regional strains, basal, midatrial and superior LA-CS, LA-LS and consequential LA-AS were increased except basal LA-CS and basal LA-AS, which proved to be impaired and unchanged, respectively. From late diastolic LA strains at atrial contraction, none of them showed any significant changes in healthy pregnant subjects as compared to that of non-pregnant cases (Table 3 and Table 4).

### 3.4. Feasibility, Post Hoc Power and Multivariable Analyses

A study involved 28 healthy pregnant women, among whom detailed LA analysis could be performed in 19 cases. Accordingly, the feasibility was 19/28, or 68%. A post hoc power analysis was performed to evaluate the statistical power of the results. Given the current sample size and a large observed effect size (Cohen’s d = 0.8), this study achieved a power of 0.68 at a significance level of α = 0.05. After adjusting for key covariates in a multiple regression analysis, including heart rate and blood pressure, body surface area was identified as the independent predictor of LA expansion.

### 3.5. Interobserver and Intraobserver Variability

For interobserver variability, the ICCs for Vmax, VpreA, Vmin, and peak LA-RS, LA-LS, LA-CS, LA-AS, and LA-3DS were 0.94, 0.85, 0.96, 0.67, 0.78, 0.68, 0.64, and 0.66, respectively (all *p* < 0.05). Intraobserver reliability showed similarly high correlations, with ICC values of 0.95, 0.88, 0.95, 0.76, 0.76, 0.59, 0.73, and 0.68, respectively (all *p* < 0.05).

## 4. Discussion

To support maternal and fetal metabolic needs, pregnancy triggers profound hemodynamic adaptations, characterized by a 40–50% expansion in both plasma volume and cardiac output by the 32nd week. This elevation in cardiac output is initially mediated by augmented SV, while an increased heart rate becomes the primary driver in later stages. Despite structural cardiac remodeling, myocardial function is typically maintained. Key systemic hallmarks include a reduction in vascular resistance and a shift toward a hypercoagulable state [1].

While real-time 3D echocardiography is an established alternative for measuring LA volumes [10], the rapidly expanding 3DSTE offers the unique advantage of simultaneously assessing volumes, functional properties, and strains from a single acquired dataset and a created virtual 3D LA cast [11,12,13,14,15]. 3DSTE has been extensively validated for the assessment of LA volumes and strains, with established normal reference ranges currently available for clinical application [6,7,8,16,17]. As a non-invasive, rapid, and cost-effective tool that requires no contrast or radiation, 3DSTE allows the detailed examination of the LA’s three distinct phases: acting as a reservoir during ventricular systole, a conduit during early diastole, and a booster pump during late diastole [2,3,4]. Current clinical evidence confirms that LA volumes provide a superior index of size compared to traditional diameters, and that LA strains carry significant diagnostic and prognostic weight [18,19]. 3DSTE provides the opportunity to assess all these parameters simultaneously, using the same acquired 3D echocardiographic dataset [5].

Evidence in the literature supports the premise that physiological pregnancy per se entails substantial volumetric and functional changes in the LA. The LA anteroposterior diameter during the second and the third trimesters is increased compared to the first trimester and the control group [20]. In women with uncomplicated twin pregnancies in the third trimester (32 weeks’ gestation), higher LA dimensions were observed [21]. At 20 weeks’ gestation, Rossi et al. observed that shifting from supine to left lateral position resulted in a significant 5% increase in LA lateral diameter and an 11% increase in LA supero-inferior diameter. At 32 weeks of gestation, LA dimensions increased significantly between the supine and the left lateral position by 15% for lateral LA diameter and by 13% for LA supero-inferior diameter. These significant increases in LA size suggest that venous return is higher in the left lateral position, an effect that is already present at 20 weeks of gestation [22]. LA area index in subjects with dyspnea was lower in healthy pregnancy [23].

LA volume and volume index increased during pregnancy, being largest postpartum [24,25]. According to Burlingame et al., LA volume increased at 18–24 weeks, 30–36 weeks, and within 48 h postpartum, but later postpartum it decreased [26]. Maximum LA volume showed an increase in the second trimester compared with that measured in the first trimester and remained unchanged in the third trimester. Minimum LA volume did not change during pregnancy in a planimetric echocardiographic study of the LA. LA fractional area change showed an increase during pregnancy from trimester to trimester [27]. It was confirmed by Ando et al. demonstrating that similarly increased LA volumes could be measured compared with controls in the second and third trimesters [28]. In contrast, (maximum) LA volume measured in systole showed a continuous increase during healthy pregnancy, which returned to the original value postpartum, as shown by Ilioeje et al. [29].

Regarding LA strains, there was no significant change in LA strain during pregnancy, but a decrease could be observed in the third trimester in a recent study [24]. In another study, in line with strain parameters, despite a gradual decrease in both LA reservoir and pump strains during pregnancy, values successfully rebounded to initial levels postpartum [30]. In a 2DSTE study assessing gravid subjects at 34–39 gestational weeks, LA antero-posterior dimensions, maximum, pre-atrial contraction and minimum LA volumes were increased in pregnant subjects compared with those of non-pregnant women. LA reservoir function, represented by LA filling volume, LA expansion index, and LA ejection fraction, was increased in pregnancy. The results obtained can be explained by the fact that both the decrease in LV compliance and the increasing heart rate had a negative influence on LA emptying. Consequently, the LA began to adaptively enlarge to maintain adequate LV filling and satisfy the increasing cardiac output requirements. LA conduit function, represented by passive LA-SV, passive LA emptying index, and LA conduit volume, was decreased during pregnancy. The reduced LA conduit function may be due to physiological myocardial hypertrophy and impaired LV relaxation. LA booster pump function, represented by active LA-SV and active LA emptying index, was increased during pregnancy. Increased LA contractility serves as a physiological response to rising LA preload and remains in a compensatory phase during normal pregnancy. Changed parameters recovered to their normal values postpartum. From strains, the peak of systolic strain, systolic strain rate and the absolute value of late diastolic strain rate increased during pregnancy, whereas the absolute value of early diastolic strain rate decreased. There was no significant difference between postpartum and control groups [31].

The present study has several implications. Firstly, it has been reaffirmed that 3DSTE is a feasible tool for the simultaneous assessment of LA volumes and strains throughout the cardiac cycle, not only in healthy individuals but also during pregnancy. Secondly, our findings indicate that among LA volumes, only the end-systolic maximum LA volume is significantly elevated in healthy women in mid-term pregnancy. In contrast, the pre-atrial contraction and end-diastolic minimum LA volumes show no significant difference compared with those of non-pregnant healthy controls. These results are in alignment with the current clinical consensus, suggesting that the pregnancy-induced increase in LA size is primarily a compensatory reservoir response to volume overload rather than a sign of diastolic impairment. However, when comparing indexed LA volumes, no significant differences were observed between the two groups. This may suggest that the increase in end-systolic LA volume is part of the adaptation to increased body weight. These results may also suggest that the observed LA enlargement is primarily related to pregnancy-induced increases in body size and/or associated changes in hemodynamic load, rather than intrinsic LA remodeling. Thirdly, among the end-systolic parameters, total LA stroke volume (TASV) and total LA emptying fraction (TAEF), as well as global, mean segmental, basal, midatrial, and superior regional LA-LSs were found to be elevated, indicating enhanced LA reservoir function. LA-CS measurements showed regional disparities. These results are in full consistency with findings reported in a recent detailed 2DSTE study in the third trimester [24]. Fourthly, elevated passive LA stroke volume (PASV) and passive LA emptying fraction (PAEF) suggest enhancement of LA conduit function during early diastole. LA volume-based functional properties and strains characterizing atrial systole did not differ between pregnant cases in the second trimester and matched non-pregnant healthy controls, suggesting unaffected LA booster pump function. This finding is in contrast with results of Naqvi et al., who reported substantial differences in maternal LA function between the second and third trimesters [24]. Our findings suggest that the adaptation process is multifaceted. In addition to longitudinal function (reflected by LA-LS), LA-CS also demonstrates significant regional alterations, which may be attributed to the functional adaptation of LA myocardial fibers. Moreover, these parameters appear to be highly dependent on the specific phase of the LA cycle during which they are measured (in end-systole vs. in end-diastole). It appears that LA alterations in the second trimester are not as pronounced as those detectable in the third trimester. To confirm this, further detailed LA volume-based and strain-based analyses are required in a longitudinal study of the same cohort, potentially incorporating other imaging modalities. At present, distinguishing between physiological and pathological LA remodeling remains challenging. However, given that several LA parameters—such as volumes and strains—have demonstrated prognostic value in both specific pathologies and healthy individuals, future studies should focus on whether these parameters play a prognostic role in maternal or fetal clinical outcomes. Furthermore, future investigations should include cases of gestational hypertension and gestational diabetes to clarify which parameters may possess prognostic value in predicting adverse clinical events.

### 4.1. Limitation Section

- A significant limitation is that the image quality produced by the 3DSTE tool used in this study is generally considered lower compared with traditional 2D echocardiography images. Due to its superior spatial and temporal resolution, 2D echocardiography maintains an advantage over 3DSTE in terms of image quality [32]. Additionally, the larger footprint of the 3DSTE probe complicates precise positioning. The multi-beat acquisition protocol (integrating six subvolumes) further introduces the risk of stitching artifacts and motion-related errors, potentially compromising the reliability of the data [11,12,13,14,15].
- The validation of 3DSTE-derived LA volumetric and strain analysis was beyond the scope of the present study, as it has already been validated, like against 2D echocardiography and volumetric 3D echocardiography in previous investigations [6,16]. Nevertheless, the scientific robustness of our findings could have been further strengthened by incorporating an alternative imaging modality for simultaneous internal validation.
- The evaluation was strictly focused on the LA; no functional or volumetric parameters of any other heart chambers were included in the analysis. The absence of LV strain or right heart evaluation limits the ability to interpret atrioventricular coupling and global cardiac adaptation.
- Due to the relatively small healthy cohort, this study may be statistically underpowered, leaving the possibility of Type II errors despite high measurement reproducibility. Consequently, these findings should be considered exploratory and require validation in larger, multi-center studies.
- The current study relies on a cross-sectional design rather than longitudinal follow-up of the same cohort, which limits our ability to definitively attribute the observed differences to pregnancy-related adaptations. This emphasizes the importance of prospective follow-up studies, which can be conducted relatively easily and within a manageable timeframe in a pregnant population.

### 4.2. Conclusions

With a detailed 3DSTE study, elevated end-systolic LA volume and preserved diastolic LA volumes together with enhanced end-systolic LA reservoir and early diastolic LA conduit functional properties could be detected with features of preserved late diastolic booster pump function in healthy women during mid-term pregnancy (second trimester). When comparing indexed LA volumes, no significant difference could be confirmed between the pregnant and non-pregnant groups. This suggests that the increased end-systolic LA volume may be an adaptation to increased body weight.

## Figures and Tables

**Table 1:** Two-dimensional echocardiographic data of pregnant and non-pregnant healthy individuals.

	Non-Pregnant Healthy Cases (n = 43)	Pregnant Healthy Subjects (n = 19)
LA diameter (mm)	35.1 ± 4.0	33.3 ± 3.3
LV end-diastolic diameter (mm)	47.0 ± 3.6	48.4 ± 3.7
LV end-diastolic volume (mL)	96.0 ± 25.2	108.7 ± 14.9
LV end-systolic diameter (mm)	31.6 ± 3.1	28.6 ± 3.4
LV end-systolic volume (mL)	33.7 ± 8.0	30.6 ± 7.2
Interventricular septum (mm)	8.3 ± 1.3	9.0 ± 0.6 *
LV posterior wall (mm)	8.7 ± 1.4	8.8 ± 0.7
LV ejection fraction (%)	65.6 ± 4.9	71.9 ± 6.2 *
E velocity (cm/s)	86.9 ± 13.3	56.2 ± 15.4 *
A velocity (cm/s)	62.0 ± 18.0	52.1 ± 11.7 *

* *p* < 0.05 versus non-pregnant healthy cases. **Abbreviations:** LA = left atrium; LV = left ventricle, E and A = transmitral E and A flow velocity.

**Table 2:** Comparison of 3DSTE-derived volumetric left atrial parameters between pregnant and non-pregnant healthy individuals.

	Non-Pregnant Healthy Cases (n = 43)	Pregnant Healthy Subjects (n = 19)
**Calculated Volumes**		
Vmax (mL)	34.9 ± 11.5	40.9 ± 6.5 *
Vmax/BSA	20.4 ± 6.7	19.4 ± 6.2
VpreA (mL)	22.4 ± 7.1	23.2 ± 6.4
VpreA/BSA	13.1 ± 4.2	10.9 ± 2.8
Vmin (mL)	15.9 ± 5.7	16.2 ± 4.7
Vmin/BSA	9.3 ± 3.3	7.8 ± 2.0
**Stroke Volumes**		
TASV (mL)	19.0 ± 7.9	24.7 ± 6.0 *
PASV (mL)	12.5 ± 6.1	17.7 ± 6.3 *
AASV (mL)	6.5 ± 3.5	7.0 ± 4.6
**Emptying fractions**		
TAEF (%)	53.5 ± 12.0	60.2 ± 10.2 *
PAEF (%)	35.0 ± 10.3	43.3 ± 12.5 *
AAEF (%)	28.6 ± 13.1	29.2 ± 12.5

* *p* < 0.05 versus non-pregnant healthy cases. **Abbreviations:** AASV = active atrial stroke volume; AAEF = active atrial emptying fraction; BSA = body surface area; PAEF = passive atrial emptying fraction; PASV = passive atrial stroke volume; TAEF = total atrial emptying fraction; TASV = total atrial stroke volume; Vmax = maximum left atrial volume; Vmin = minimum left atrial volume; VpreA = left atrial volume before atrial contraction.

**Table 3:** Comparison of 3DSTE-derived global, mean segmental and regional peak left atrial strain parameters between pregnant and non-pregnant healthy individuals.

	Non-Pregnant Healthy Cases (n = 43)	Pregnant Healthy Subjects (n = 19)
**Global**		
**RS (%)**	−13.2 ± 8.5	−15.6 ± 9.1
**CS (%)**	34.3 ± 15.5	39.4 ± 15.4
**LS (%)**	25.6 ± 9.1	41.5 ± 9.2 *
**3DS (%)**	−5.7 ± 5.7	−7.8 ± 4.8
**AS (%)**	68.1 ± 27.5	100.9 ± 35.4 *
**Mean segmental**		
**RS (%)**	−17.6 ± 7.0	−19.8 ± 8.1
**CS (%)**	38.2 ± 14.9	42.8 ± 14.5
**LS (%)**	28.9 ± 8.5	42.4 ± 9.0 *
**3DS (%)**	−11.0 ± 5.1	−12.2 ± 5.0
**AS (%)**	73.7 ± 27.5	103.8 ± 34.6 *
**Regional**		
**RS _basal_ (%)**	−18.1 ± 9.3	−17.9 ± 7.5
**RS _midatrial_ (%)**	−17.6 ± 8.3	−20.9 ± 9.8
**RS _superior_ (%)**	−17.5 ± 12.1	−20.9 ± 11.0
**CS _basal_ (%)**	40.8 ± 14.5	31.1 ± 10.4 *
**CS _midatrial_ (%)**	32.5 ± 12.6	40.4 ± 12.9 *
**CS _superior_ (%)**	42.5 ± 30.5	64.1 ± 30.4 *
**LS _basal_ (%)**	24.0 ± 12.3	31.0 ± 9.1 *
**LS _midatrial_ (%)**	34.5 ± 13.0	54.5 ± 9.8 *
**LS _superior_ (%)**	25.6 ± 14.8	41.5 ± 18.2 *
**3DS _basal_ (%)**	−11.9 ± 7.3	−13.1 ± 5.7
**3DS _midatrial_ (%)**	−10.3 ± 5.9	−12.1 ± 6.5
**3DS _superior_ (%)**	−11.0 ± 9.5	−11.0 ± 6.5
**AS _basal_ (%)**	63.9 ± 22.4	64.2 ± 19.5
**AS _midatrial_ (%)**	71.8 ± 26.0	111.2 ± 29.0 *
**AS _superior_ (%)**	88.7 ± 78.0	152.2 ± 84.0 *

*p < 0.05 versus controls. **Abbreviations:** RS = radial strain; CS = circumferential strain; LS = longitudinal strain; 3DS = three-dimensional strain; AS = area strain.

**Table 4:** Comparison of 3DSTE-derived global, mean segmental and regional left atrial strain parameters at atrial contraction between pregnant and non-pregnant healthy individuals.

	Non-Pregnant Healthy Cases (n = 43)	Pregnant Healthy Subjects (n = 19)
**Global**		
**RS (%)**	−4.2 ± 5.9	−6.5 ± 5.6
**CS (%)**	11.9 ± 11.3	13.6 ± 8.9
**LS (%)**	9.1 ± 8.8	8.4 ± 7.3
**3DS (%)**	−2.4 ± 5.4	−4.0 ± 4.3
**AS (%)**	20.8 ± 22.3	23.5 ± 19.1
**Mean segmental**		
**RS (%)**	−6.7 ± 4.9	−8.2 ± 4.3
**CS (%)**	14.1 ± 9.5	17.1 ± 7.4
**LS (%)**	9.6 ± 5.5	10.9 ± 5.9
**3DS (%)**	−4.2 ± 4.6	−5.9 ± 4.0
**AS (%)**	24.0 ± 16.6	30.9 ± 13.8
**Regional**		
**RS _basal_ (%)**	−7.2 ± 6.3	−8.1 ± 3.9
**RS _midatrial_ (%)**	−6.1 ± 5.0	−8.3 ± 5.1
**RS _superior_ (%)**	−6.7 ± 7.8	−8.1 ± 5.4
**CS _basal_ %)**	14.3 ± 9.3	14.9 ± 7.8
**CS _midatrial_ (%)**	12.1 ± 9.7	15.3 ± 8.5
**CS _superior_ (%)**	16.8 ± 16.0	23.3 ± 12.8
**LS _basal_ (%)**	7.3 ± 5.3	9.1 ± 4.0
**LS _midatrial_ %)**	11.4 ± 8.4	11.8 ± 10.5
**LS _superior_ (%)**	10.4 ± 9.2	12.3 ± 7.7
**3DS _basal_ (%)**	−4.5 ± 5.7	−6.0 ± 3.8
**3DS _midatrial_ (%)**	−3.5 ± 4.6	−5.8 ± 4.7
**3DS _superior_ %)**	−4.9 ± 8.4	−5.9 ± 5.2
**AS _basal_ (%)**	20.3 ± 14.7	24.2 ± 12.0
**AS _midatrial_ (%)**	23.8 ± 17.3	28.6 ± 21.1
**AS _superior_ (%)**	29.9 ± 31.4	44.3 ± 27.3

Abbreviations: RS = radial strain; CS = circumferential strain; LS = longitudinal strain; 3DS = three-dimensional strain; AS = area strain.

## Data Availability

The data presented in this study are available on request from the corresponding author. The data are not publicly available due to local restrictions.

## References

[1] Regitz-Zagrosek V., Roos-Hesselink J.W., Bauersachs J., Blomström-Lundquist C., Cífková R., De Bonis M., Iung B., Johnson M.R., Kintscher U., Kranke P. (2018). 2018 ESC Guidelines for the management of cardiovascular diseases during pregnancy. Eur. Heart J..

[2] Hoit B.D. (2014). Left atrial size and function: Role in prognosis. J. Am. Coll. Cardiol..

[3] Blume G.G., Mcleod C.J., Barnes M.E., Seward J.B., Pellikka P.A., Bastiansen P.M., Tsang T.S.M. (2011). Left atrial function: Physiology, assessment, and clinical implications. Eur. J. Echocardiogr..

[4] Seward J.B., Hebl V.B. (2014). Left atrial anatomy and physiology: Echo/Doppler assessment. Curr. Opin. Cardiol..

[5] Domsik P., Kalapos A., Chadaide S., Sepp R., Hausinger P., Forster T., Nemes A. (2014). Three-dimensional speckle tracking echocardiography allows detailed evaluation of left atrial function in hypertrophic cardiomyopathy-insights from the MAGYAR-Path Study. Echocardiography.

[6] Nemes A., Domsik P., Kalapos A., Lengyel C., Orosz A., Forster T. (2014). Comparison of three-dimensional speckle tracking echocardiography and two-dimensional echocardiography for evaluation of left atrial size and function in healthy volunteers (results from the MAGYAR-Healthy Study). Echocardiography.

[7] Nemes A., Kormányos Á., Domsik P., Kalapos A., Lengyel C., Forster T. (2019). Normal reference values of three-dimensional speckle-tracking echocardiography-derived left atrial strain parameters (results from the MAGYAR-Healthy Study). Int. J. Cardiovasc. Imaging.

[8] Nemes A., Kormányos Á., Domsik P., Kalapos A., Ambrus N., Lengyel C. (2021). Normal reference values of left atrial volumes and volume-based functional properties using three-dimensional speckle-tracking echocardiography in healthy adults (Insights from the MAGYAR-Healthy Study). J. Clin. Ultrasound.

[9] Lang R.M., Badano L.P., Mor-Avi V., Afilalo J., Armstrong A., Ernande L., Flachskampf F.A., Foster E., Goldstein S.A., Kuznetsova T. (2015). Recommendations for cardiac chamber quantification by echocardiography in adults: An update from the American Society of Echocardiography and the European Association of Cardiovascular Imaging. Eur. Heart J. Cardiovasc. Imaging.

[10] Anwar A.M., Soliman O.I.I., Geleijnse M.L., Nemes A., Vletter W.B., ten Cate F.J. (2008). Assessment of left atrial volume and function by real-time three-dimensional echocardiography. Int. J. Cardiol..

[11] Franke A., Kuhl H.P. (2003). Second-generation real-time 3D echocardiography: A revolutionary new technology. MedicaMundi.

[12] Ammar K.A., Paterick T.E., Khandheria B.K., Jan M.F., Kramer C., Umland M.M., Tercius A.J., Baratta L., Tajik A.J. (2012). Myocardial mechanics: Understanding and applying three-dimensional speckle tracking echocardiography in clinical practice. Echocardiography.

[13] Urbano-Moral J.A., Patel A.R., Maron M.S., Arias-Godinez J.A., Pandian N.G. (2012). Three-dimensional speckle-tracking echocardiography: Methodological aspects and clinical potential. Echocardiography.

[14] Muraru D., Niero A., Rodriguez-Zanella H., Cherata D., Badano L. (2018). Three-dimensional speckle-tracking echocardiography: Benefits and limitations of integrating myocardial mechanics with three-dimensional imaging. Cardiovasc. Diagn. Ther..

[15] Gao L., Lin Y., Ji M., Wu W., Li H., Qian M., Zhang L., Xie M., Li Y. (2022). Clinical Utility of Three-Dimensional Speckle-Tracking Echocardiography in Heart Failure. J. Clin. Med..

[16] Kleijn S.A., Aly M.F.A., Terwee C.B., van Rossum A.C., Kamp O. (2011). Comparison between direct volumetric and speckle tracking methodologies for left ventricular and left atrial chamber quantification by three-dimensional echocardiography. Am. J. Cardiol..

[17] Mochizuki A., Yuda S., Oi Y., Kawamukai M., Nishida J., Kouzu H., Muranaka A., Kokubu N., Shimoshige S., Hashimoto A. (2013). Assessment of left atrial deformation and synchrony by three-dimensional speckle-tracking echocardiography: Comparative studies in healthy subjects and patients with atrial fibrillation. J. Am. Soc. Echocardiogr..

[18] Javadi N., Bismee N.N., Abbas M.T., Scalia I.G., Pereyra M., Ali N.B., Esfahani S.A., Awad K., Farina J.M., Ayoub C. (2024). Left Atrial Strain: State of the Art and Clinical Implications. J. Pers. Med..

[19] Pritchett A.M., Jacobsen S.J., Mahoney D.W., Rodeheffer R.J., Bailey K.R., Redfield M.M. (2003). Left atrial volume as an index of left atrial size: A population-based study. J. Am. Coll. Cardiol..

[20] Wang X.-J., Chen L.-L., Hong M.-H., Kong L.-Y., Xiang W., Fu L., Li X.-W., Liu F. (2025). Dynamic change in maternal cardiac function during pregnancy. Front. Cardiovasc. Med..

[21] Toncelli L., Pasquini L., Masini G., Orlandi M., Paci G., Mecacci F., Pedrizzetti G., Galanti G. (2022). Difference in cardiac remodeling between female athletes and pregnant women: A case control study. Cardiovasc. Ultrasound.

[22] Rossi A., Cornette J., Johnson M.R., Karamermer Y., Springeling T., Opic P., Moelker A., Krestin G.P., Steegers E., Roos-Hesselink J. (2011). Quantitative cardiovascular magnetic resonance in pregnant women: Cross-sectional analysis of physiological parameters throughout pregnancy and the impact of the supine position. J. Cardiovasc. Magn. Reson..

[23] Mostafavi A., Feizian M., Fotook Kiaei S.Z., Tabatabaei S.A. (2022). Dyspnea in pregnancy might be related to the incomplete physiological adaptation of the heart. J. Cardiovasc. Thorac. Res..

[24] Naqvi T.Z., Narayanan M., Rafie R., Qamruddin S., Lee M.S., Girardo M.E., Daneshvar S., Wen S., Stek A.M., Elkayam U. (2024). Cardiovascular Adaptation in Normal Pregnancy with 2D and 3D Echocardiography, Speckle Tracking, and Radial Artery Tonometry. JACC Adv..

[25] Dobrowolski P., Kosinski P., Prejbisz A., Szczepkowska A., Klisiewicz A., Januszewicz M., Wielgos M., Januszewicz A., Hoffman P. (2021). Longitudinal changes in maternal left atrial volume index and uterine artery pulsatility indices in uncomplicated pregnancy. Am. J. Obstet. Gynecol..

[26] Burlingame J.M., Yamasato K., Ahn H.J., Seto T., Wilson Tang W.H. (2017). B-type natriuretic peptide and echocardiography reflect volume changes during pregnancy. J. Perinat. Med..

[27] Valensise H., Novelli G.P., Vasapollo B., Borzi M., Arduini D., Galante A., Romanini C. (2000). Maternal cardiac systolic and diastolic function: Relationship with uteroplacental resistances. A Doppler and echocardiographic longitudinal study. Ultrasound Obstet. Gynecol..

[28] Ando T., Kaur R., Holmes A.A., Brusati A., Fujikura K., Taub C.C. (2015). Physiological adaptation of the left ventricle during the second and third trimesters of a healthy pregnancy: A speckle tracking echocardiography study. Am. J. Cardiovasc. Dis..

[29] Iloeje U.N., Jesurobo D., Mankwe A.C., Kweki A.G., Aiwujo H.O., Oladimeji O.M., Emenena I., Akpa M.R., Odia O.J. (2023). Cardiac Dimensions in Normal Pregnancy: A Prospective Study. Cureus.

[30] Tasar O., Kocabay G., Karagoz A., Karabay A.K., Karabay C.Y., Kalkan S., Kirma C. (2019). Evaluation of Left Atrial Functions by 2-dimensional Speckle-Tracking Echocardiography During Healthy Pregnancy. J. Ultrasound Med..

[31] Song G., Liu J., Ren W., Qiao W., Zhang J., Zhan Y., Bi W. (2015). Reversible Changes of Left Atrial Function during Pregnancy Assessed by Two-Dimensional Speckle Tracking Echocardiography. PLoS ONE.

[32] Arslan A., Ilis D., Artac I., Karakayali M., Omar T., Erata Y., Karabag Y., Rencuzogullari I. (2025). Relationship Between Left Atrial Coupling Index and Atrial High-Rate Episodes. Pacing Clin. Electrophysiol..

